# Human skin explant model for the investigation of topical therapeutics

**DOI:** 10.1038/s41598-020-78292-4

**Published:** 2020-12-03

**Authors:** Jessica E. Neil, Marc B. Brown, Adrian C. Williams

**Affiliations:** 1 Research Triangle Park, Durham, NC USA; 2 MedPharm Ltd, Guildford, UK; 3 University of Reading, Reading, UK; 4 MedPharm Ltd, 4222 Emperor BLVD, Suite 320, Durham, NC 27703 USA

**Keywords:** Biological techniques, Biotechnology

## Abstract

The development of in vitro and ex vivo models to mimic human illness is important not only for scientific understanding and investigating therapeutic approaches but also to mitigate animal testing and bridge the inter-species translational gap. While in vitro models can facilitate high-throughput and cost-efficient evaluation of novel therapeutics, more complex ex vivo systems can better predict both desirable and adverse in vivo effects. Here we describe an ex vivo cultured human skin explant model in which we have characterized pathological tissue integrity, barrier function and metabolic stability over time. Our findings suggest that human skin can be successfully cultured for pharmacodynamic use up to and beyond 9 days without any adverse physiological consequence.

## Introduction

The testing of topical cosmetic and pharmaceutical excipients and active ingredients for possible irritation effects and potency has slowly evolved since its inception in the early 1900s. In 1944, the first U.S. Food and Drug Administration recognized animal testing was implemented; known as the “Draize test” after toxicologist Dr. John Henry Draize, it utilized rabbits as test subjects for ocular and skin irritation of cosmetics and personal care items. While advances in animal testing allowed a decrease from 6 to 1–3 rabbits per test, researchers, consumers and pressure groups refuted the need and value of such tests, and ultimately government legislation is moving to ban animal testing for topical products. The 7th amendment to the European Union Cosmetics Directive now forbids animal testing of cosmetics in Europe^1^. Notably, the dissimilarities between animal and human tissue raises questions regarding the legitimacy of data that is generated; for skin, these differences include the thickness of the tissue itself and its constituent layers, density of the hair follicles and other appendages, and immune system irregularities.

In 1988 the European Centre for the Validation of Alternative Methods (ECVAM) Skin Irritation Task Force published a report evaluating new in vitro testing methods classifying skin irritants^2^. From this report it was suggested that monolayer keratinocyte cultures, while useful, lack the inherent barrier function and properties of skin and in particular its outermost stratum corneum, making them unsuitable for routine testing of chemicals for skin irritation. From the 1980′s, reconstituted human epithelium (RHE) skin models were developed, in part for human irritancy studies. These cultures allowed differentiation of an intact stratum corneum in an air–liquid interface that more closely resembled the in vivo human skin barrier. These models quickly gained interest for pharmacotoxicology studies and dermal irritation evaluation with commercially produced systems such as EpiSkin (L’Oreal) and EpiDerm (MatTek Corporation) available. However, these models still lack immune-associated cell types, either resident or carried by vascularisation, that are present with in vivo human skin clinical studies.

In addition to the prominent keratinocytes which can produce antimicrobial peptides such as psoriasin, lipocalin and defensins, immune cells play a major role in the pathophysiology of skin disorders. In the absence of cell migration from the circulatory and lymph system into the dermis, resident immune cells remain; lymphocytes, dendritic cells and Langerhans cells. Of these, the resident memory T cells are known to be potent mediators against infection, but also function in autoimmune disease. There are approximately 20 billion memory T cells in the skin of an adult human, with an estimated 1 million in each cm^2^^3–5^. To further elucidate the function of these resident T cells, mitogen stimulation was used to determine the polarization of the cells via secreted cytokines and surface markers. It was found that the predominant skin resident T cell population secreted Th1 cytokines IFN-γ and IL-2, with a small sample also producing the Th2 cytokine IL-4^5^.

Organ culture of human skin dates back over 50 years^6^ and has undergone significant improvements to optimize the tissue for research studies developing therapeutics and formulations without the need for animal testing. The ability to maintain a Human Explant Skin Culture (HESC) in submerged versus air–liquid interface cultures and the effects of cytokine stimulation have been studied^7^ and more recently the viability of HESC at an air–liquid interface was shown for up to 4 weeks in culture^8^. The benefits of using HESC cultures over reconstructed human epithelial models (RHE) are evident from varied studies investigating the effects of environmental stress, aging and skin diseases^1^ and as a model for wound healing^9^. Cytokine stimulation of HESC provoked immune cell responses with release of chemokines, antimicrobial peptides and keratinocyte differentiation markers associated with clinical psoriasis^10^. However, in the HESC model literature^1,6,11–13^, a general increase in epidermal thickness is noted over the first week in culture with a decrease in tissue integrity and keratinocyte differentiation occurring after ~ 14 days. In addition, a loss of keratinocyte proliferation is accompanied by an increase in keratinocyte apoptosis evident after the first week^9^. In contrast, the epithelial barrier function was reportedly intact up to 28 days in culture, when assessed using topically applied transgene expression^8^. Given the growing interests and need for HESC models for toxicity, irritation, and therapeutic efficacy testing across diverse disciplines including the pharmaceutical, cosmetic and agrochemical sectors, here we assess tissue viability, mechanical barrier properties and metabolic activity of HESC with time in culture.

## Results and discussion

### Assessment of skin viability over time

**Figure 1:**
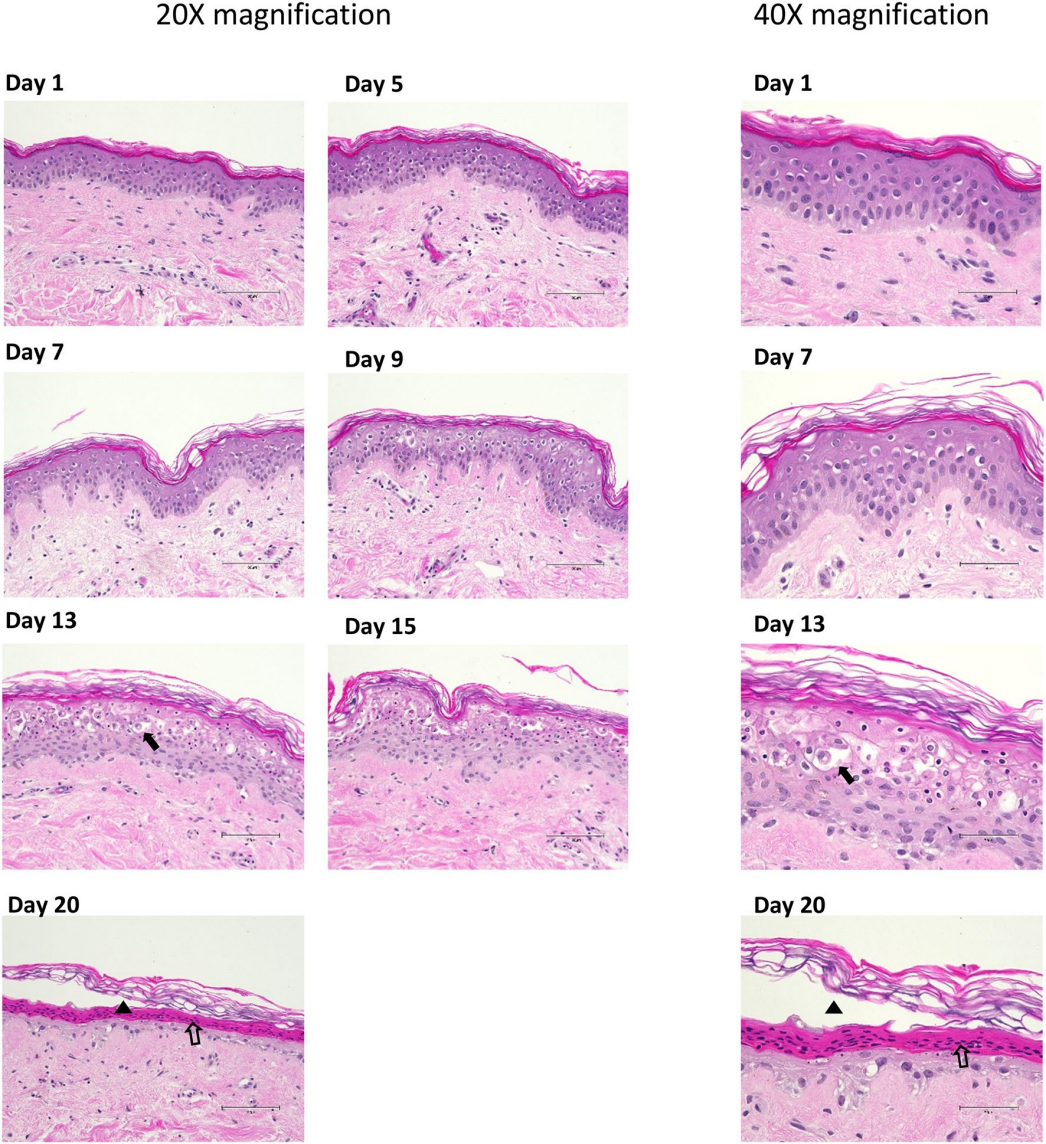
Haematoxylin and eosin staining of tissue integrity over 20 days observing spongiosis, necrosis, parakeratosis and epidermal/dermal separation. Representative images from Donor 1 haematoxylin and eosin stained tissue over time course. Days 1, 5, 7, 9, 13, 15 and 20 exhibited at × 20 magnification (scale bar = 100 µm). Days 1, 7, 13 and 20 exhibited at × 40 magnification (scale bar = 50 µm). Solid arrow points to spongiosis (Day 13). Empty arrow points to necrosis (Day 20). Solid triangle points to parakeratosis (Day 20).

**Figure 2:**
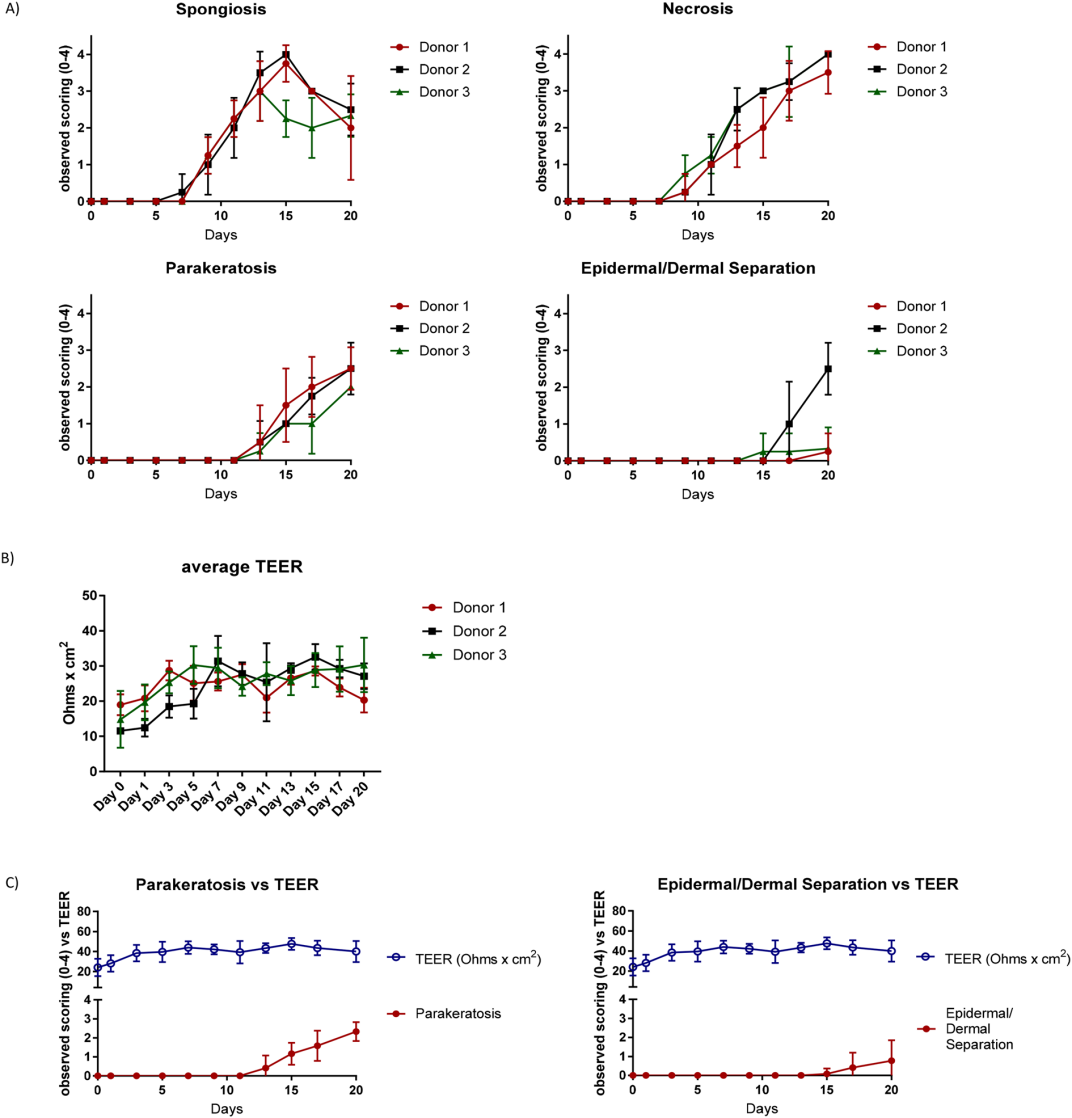
(**A**) The change in trans-epithelial electrical resistance (TEER) as a measure of barrier integrity. Average TEER measurement over time in culture. N = 3 donors, 4 replicates per donor per time point. Reported TEER as Ohms*cm^2^ tissue surface area. Error bars represent mean ± standard deviation. (**B**) Histological analysis of tissue with haematoxylin/eosin staining over time course. Average score measurement over time in culture. N = 3 donors, 4 replicates per donor per time point. Error bars represent mean ± standard deviation. (**C**) Ratio of parakeratosis and epidermal/dermal separation to TEER. Comparison of average score measurement of parakeratosis and epidermal/dermal separation over time in culture versus reported TEER as Ohms*cm^2^ tissue surface area. N = 3 donors, 4 replicates per donor per time point. Error bars represent mean ± SD. Scoring values were analysed using unpaired non-parametric two-tailed Mann Whitney t-test with GraphPad Prism software.

To determine tissue integrity over the 20-day time course in culture, haematoxylin and eosin staining of the tissue cross-section was performed (Fig. 1). Images were scored for spongiosis, necrosis, parakeratosis and epidermal/dermal separation (Fig. 2). Potential thickening of the epidermal layer (hyperplasia) was measured but no increase in epidermal thickness was observed over this period (data not shown).

The appearance of vacuolar fluid-filled cells in the epidermis indicates that spongiosis occurred on Day 9 in culture and steadily increased in prevalence up to day 15 (*P* < 0.0001). Thereafter, necrosis of the keratinocytes diminished the appearance of spongiosis. (Fig. 2A). Necrosis (determined by condensation of nuclei and bright red staining) was first apparent at Day 9 in culture and increased up to day 20 (*P* < 0.0001). Parakeratosis, the thickening and sloughing of the stratum corneum was also a later event first observed on day 13 in culture. While an increase in parakeratosis was observed, it was not uniform across the whole sample with approximately 60% of the tissue area affected by day 20 (*P* < 0.0001). Finally, separation of the epidermal and dermal layers is attributable to disintegration of the dermo-epidermal junction (basement membrane) and again was not uniform across the whole sample but extended to ca. 20% of the total tissue area by day 20 (*P* = 0.0211). In combination, the histological analysis demonstrates that tissue integrity is maintained unimpaired for up to 9 days in culture under the described conditions.

To assess skin barrier integrity, Trans-Epithelial Electrical Resistance (TEER) is commonly measured^14^. Based on this approach electrical resistance can be quantified using an alternating current (AC) across the membrane to observe the integrity of tight junctions and involvement of any barrier dysfunction (Fig. 2B). Combined donors resulted in a 53.5% ± 16.5 increase from Day 0 (day of fresh tissue harvest) to Day 3 in culture. This change in TEER increased to 101% ± 68.5 by Day 7.

TEER readings are correlated with observed barrier disfunction of parakeratosis and epidermal/dermal separation in Fig. 2C. The increase in parakeratosis and epidermal/dermal separation observed is not mirrored by the loss of TEER measured resistance beginning on Day 13. However, it is evident that the histological scoring captured morphological dysfunction earlier than Day 13, suggesting that TEER cannot detect early tissue integrity loss, though electrical resistance measurements remain useful for assessing gross barrier integrity of thinner tissue samples, such as those used for in vitro permeation studies.

### Tissue metabolic activity

**Figure 3:**
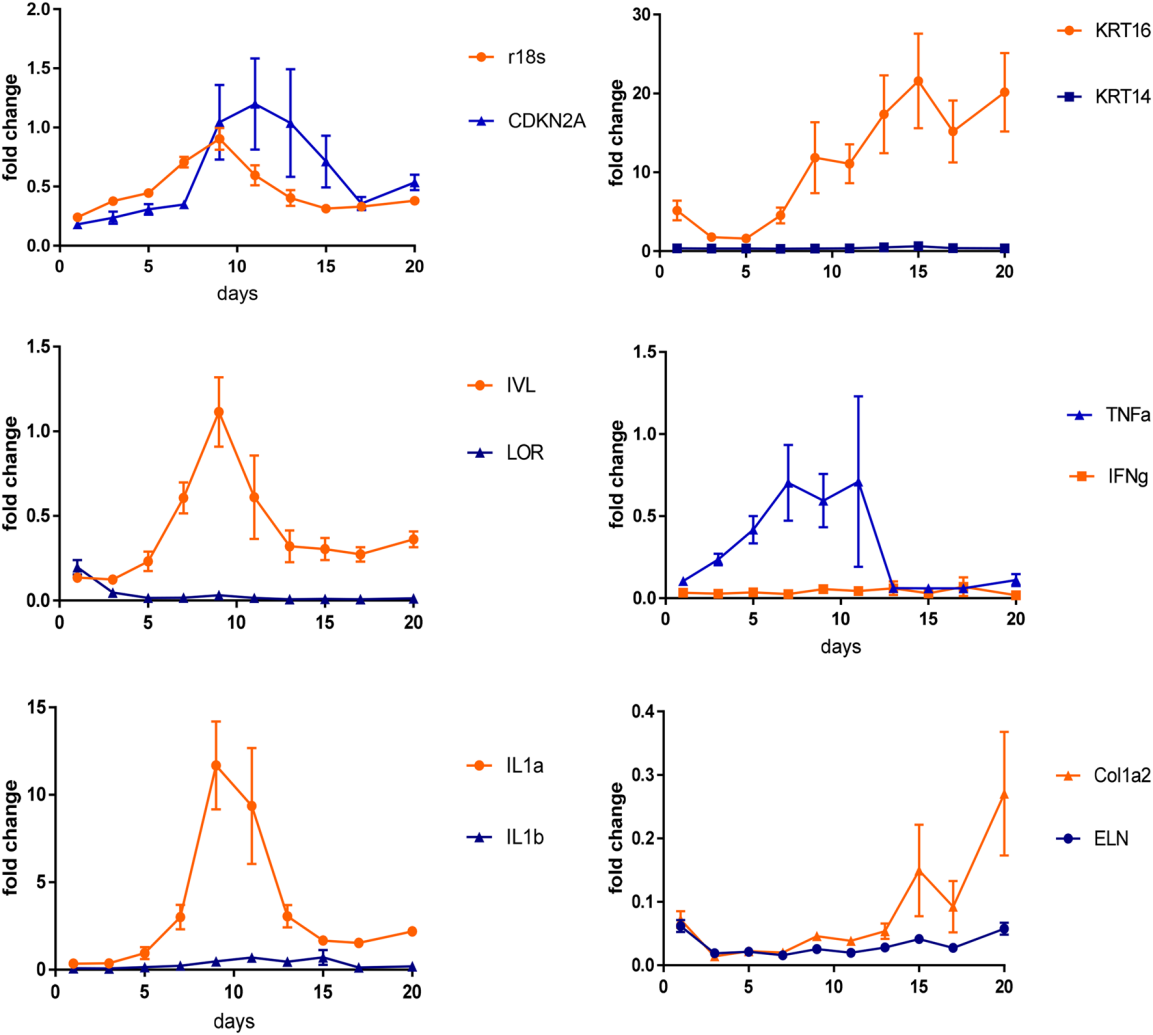
Tissue metabolic activity measured by gene expression RT-qPCR. Gene expression displayed as fold change compared to Day 0. N = 3 combined donors; 4 samples per time point. Error bars represent mean ± standard error of the mean.

To assess cell viability and metabolic capacity over time, RT-qPCR of common housekeeping, cell cycle and proliferation-associated genes was performed (Fig. 3). The housekeeping genes ribosomal 18s (r18S), a ribosomal subunit integral in all protein translation within the eukaryotic cell, and cyclin-dependent kinase inhibitor 2A (CDKN2A), a biomarker of cellular senescence were compared^15^. Gene expression of r18s increases gradually from Day 1 to Day 9 (ca. 3.7 fold; *P* < 0.0001), suggesting an increase of cellular metabolism. However, on Day 9 a spike in CDKN2A mRNA was observed (ca. 5.7 fold; *P* = 0.2145), followed by an immediate arrest of r18s increase, suggesting a switch in the tissue metabolic paradigm.

Keratin 14 is a commonly used biomarker of dividing basal keratinocytes and tends to decrease as the cells differentiate and migrate to the stratified epithelium^16^. Keratin 16 is constitutively expressed at low levels in the epithelium but expression is induced upon injury^17^. Here, using healthy adult human skin, the expression of KRT14 remained at low levels and did not change throughout the tissue culture period suggesting no induction of keratinocyte proliferation. In contrast, the wound indicative expression of KRT16 rose steadily from Day 5 in culture reaching maximal levels at Day 15 (ca. 13.3 fold; *P* = 0.0043).

Involucrin (IVL) gene expression occurs after the maturing keratinocytes have left the basal layer but before the onset of envelope cross-linking in the upper epidermal layers and so IVL is a good biomarker for early keratinocyte differentiation^18^. Loricrin (LOR) expression is a later stage marker of keratinocyte terminal differentiation as a major component in the cornified barrier structure^19^. The observed gradual increase in IVL expression from Day 1 to Day 9 (ca. 8.2 fold; *P* < 0.0001) suggests a moderate return to keratinocyte maturation in the healthy tissue with a lack of terminal differentiation upregulation given the absence of late state LOR expression.

Matrix metalloproteinases function in tissue wound repair by facilitating extracellular matrix remodelling through the degradation of collagens, elastin, laminin and fibronectin^20^. Matrix metalloproteinase 9 (MMP9) is classified as a gelatinase prevalent in acute and chronic wounds and secreted primarily by migrating keratinocytes^21^. Alternatively, matrix metalloproteinase 12 (MMP12) is classified as an elastase specifically expressed by macrophages^22^. MMP12 showed a fluctuating pattern of expression in the HESC with high variability, while MMP9 expression was not induced over the tissue culture time course (data not shown).

A major component of skin integrity is regulation of the inflammatory profile. Tumour necrosis factor alpha (TNFα) is a prevalent cytokine in tissue homeostasis. As a cellular signalling factor, TNFα can mediate cellular apoptosis, survival, proliferation and differentiation and regulation of TNFα levels is critical for proper tissue viability^23^. In contrast to its accepted anti-pathogenic properties, in vitro studies have shown the ability of TNFα to stimulate fibroblast proliferation^24^. Alternatively, TNFα can inhibit the proliferation of keratinocytes without inducing cell death^25^. While it is difficult to elucidate the exact function of the increase in TNFα in the HESC from day 5 to day 11 in culture (ca. 1.7 fold; *P* = 0.9856), the lack of accompanying proliferation marker KRT14 and no observed necrosis by histology in this time frame suggest that survival may be one of the effects. Interferon-gamma (IFNγ) is a cytokine released by lymphocytes in response to antigen. No increase in IFNγ was observed suggesting a lack of unspecific T cell activation over the time course^26^.

Interleukin 1α (IL1α) is produced by keratinocytes in the epidermis at higher levels than in any other tissue. It regulates keratinocyte differentiation and serves as an early signalling mediator of tissue damage^27^. Upon the release of IL1α due to stress or injury, a paracrine feedback loop consisting of the IL1α protein binding the IL1α receptors on the keratinocyte surface leads to the expression of additional IL1α mRNA^28^. Along with this function, IL1α is involved in wound healing, leukocyte recruitment and the induction of downstream cytokines such as TNFα, IL6 and IL8^29^. In our study, the increase in IL1α gene expression up to Day 9 in culture (ca. 34 fold; *P* < 0.0001) could be explained by the initial release of IL1α protein upon tissue damage during surgery, and the subsequent feedback loop described to induce additional mRNA expression. This also corresponds with the TNFα induction and suggests a correlation between the two cytokines. Unlike the constitutively expressed IL1α, interleukin 1ß is an induced inflammatory mediator secreted mainly by monocytes and macrophages in response to microbial infiltration and Toll-like Receptor activation and was not induced^30^.

The most abundant extra-cellular matrix component is type I collagen (Col1a2)^31^. The gradual increase in Col1a2 expression beginning at day 13 up to Day 2 (ca. fivefold; *P* = 0.0050) could be in response to the loss of tissue integrity demonstrated by increased spongiosis and parakeratosis of the epidermal layers, causing an increase in collagen production as the wound healing mechanism. While elastin expression is typically low in the dermis of adult tissue, it can be upregulated by the overexpression of IL1ß^32^. The lack of ELN upregulation corresponds to the lack of IL1ß expression.

## Conclusions

In conclusion, while some reports describe the cellular and morphological similarities and changes observed with human ex vivo skin culture, here we characterize the principle metabolic gene expression changes and correlate with the gross morphological changes to the tissue. Skin barrier integrity (as measured by TEER) could be maintained up to and possibly beyond 20 days in culture but spongiosis, necrosis and parakeratosis showed morphological dysfunction prior to a loss in TEER barrier resistance.

Gene expression of critical biomarkers provides a comprehensive assessment of the metabolic challenges that the tissue experiences during culture. The housekeeping genes r18s and CDKN2A suggest a switch in metabolic activity on day 9. No induction of keratinocyte proliferation by KRT14 gene expression was quantified, however an increase in KRT16 gene expression was observed. In contrast, keratinocyte maturation quantified by IVL continued in the HESC, but terminal differentiation was not detected by LOR expression. While the expression of these biomarkers confirms cellular metabolic activity, there is evidence of a discrepancy between HESC and skin in vivo, requiring consideration when using this model as a test platform. A primary concern in the culture of human tissue is activation of the inflammatory cytokines which can be detrimental to cellular integrity over time. TNFα and IL1α gene expression was observed suggesting a response to the wounding and subsequent recovery, however the lack of additional expression of IFNγ and IL1ß shows minimal inflammatory reaction. Lastly the collagen extracellular matrix response also supports a wound healing mechanism in these cultures though additional characterization of keratinocyte proliferation and maturation is merited.

This model augments on the previous work done by Vostálová et al. whom described disinfecting fresh tissue and the basis for the culture medium, however differs in the use of porous membrane substrates to maintain an air–liquid interface. This approach was also adapted to allow for the mounting of tissue explants into Franz cells for the application of topical formulations without concern for lateral drug migration into the media. The data demonstrates that the HESC is structurally viable and metabolically active for up to 9 days in culture and can be employed for preclinical testing of delivery and efficacy of skin therapeutics under these conditions.

## Materials and methods

### Human skin explant

All human tissue was obtained via elective abdominoplasty with donor consent under Pearl IRB approval in accordance with FDA 21 CFR 56.104 and DHHS 45 CFR 46.101 regulations (Pearl Pathways. Exemption Determination Submission. IRB Study Number: 15-MEDP-101. Study Title: Healthy volunteer skin donation for in vitro experimentation). Written informed consent was obtained from all subjects or, if subjects are under 18, from a parent and/or legal guardian. All donors were healthy and not currently taking any form of systemic corticosteroid treatment. No identifying information beyond ethnicity and age were provided, when available. Tissue was maintained in humidified incubators at 37 °C and 5% CO_2_ in either Franz cells or Costar Transwell Permeable Support (Thermo Fisher Scientific; #3470). Tissue was kept chilled until processing for culture (between 16 and 24 h post-removal) and processed for use within 24 h of surgery. Tissue was defatted and dermatomed to a thickness of 750 ± 100 µm (Integra Padgett Slimline SB). Tissue exhibiting abnormalities such as edema, abrasion, or heavy striation were discarded. Tissue was further cut into 1cm^2^ sections for mounting onto Franz cells or a 7 mm punch biopsy was used for transwell studies. The well was then filled with modified Cornification media resulting in an air–liquid interface explant culture. Media was changed no less then every 48 h. Human tissue explants were cultured in a modified Cornification media^12,33^.

### Tissue integrity time course

Human tissue explants were plated onto static Franz cells and supplemented with modified Cornification media in a humidified incubator to be harvested at each respective time point. A total of three individual donors were assayed, four replicates per donor per time point. Eleven time points were harvested; Day 0, 1, 3, 5, 7, 9, 11, 13, 15, 17 and day 20. Day 0 explants were cultured 2 h prior to harvest. At time of harvest, Cornification media was aspirated from the receptor chamber and replaced with pre-warmed 37 °C phosphate buffered saline (PBS). The donor chamber was filled with pre-warmed 37 °C PBS. Transepithelial Electrical Resistance (TEER) measurements were conducted using a Iso-Tech LCR821 Meter set at 0.0100 kHz Frequency and 0.200 Voltage. A single reading was recorded per tissue explant at each time point of harvest, as well as a Day 0 compromised explant for reference. TEER values are reported as Ohm (Ω) × cm^2^. Immediately after TEER measurement, PBS was aspirated from tissue and explant was removed from Franz cell and placed on a clean cutting board for dissection. Using a single edge blade, one half of the tissue was excised and placed in 10% Normal Buffered Formalin (NBF) for 48 h at room temperature for fixation. After fixation in 10% NBF, the section was transferred to 70% ethanol for storage until histology processing. The remaining half of explant was immediately placed into a pre-chilled Eppendorf tube and stored at − 80 °C for future use.

### Histology

Briefly, tissue was dehydrated in increasing concentrations of ethanol to xylene, then embedded in paraffin blocks and sectioned to a thickness of 5 µm and mounted on glass slides, 2 sections per slide. N = 3 donors, 4 replicates per donor per time point. The sections were then stained using haematoxylin and eosin (H&E) per suppliers’ protocol.

Tissue integrity over the time course was observed by brightfield microscopy using an Olympus CKX53 microscope and 4X, 10X, 20X and 40X objectives. A scoring parameter (0–4) was assigned for five integrity observations; vacuoles/spongiosis, dermal/epidermal separation, necrosis, parakeratosis, and epithelial hyperplasia. A score of 0 designates no observation, 1 designates observation less than < 20%, 2 designates observation between 20 and 40%, 3 designates observation between 40 and 60%, 4 designates observation of greater than 60% of overall surface area. Objective scoring was conducted by assigning observed ranges in two serial sections of tissue per sample and taking the average of the score. Scoring values were analysed using unpaired non-parametric two-tailed Mann Whitney t-test with GraphPad Prism software. Representative images taken using Thermo EVOS M5000 microscope and 20 × and 40 × objectives.

### RNA isolation and RT-qPCR

Human skin explant samples were stored in RNALater (Invitrogen AM7021) to allow permeation overnight at 4 °C before Isolate RNA was used per Qiagen RNeasy Mini Kit (Qiagen 74106) instructions. Reverse transcription used a High Capacity cDNA kit (Applied Biosystems 4368814). RT-qPCR was run on an Applied Bioscience QuantStudio 6 Flex Real-Time PCR System. All primers were commercially available and purchased from Invitrogen Life Technologies. Fold change was calculated as ratio of the power of the gene of interest divided by the average power of the samples at Day 0. Statistical testing used GraphPad Prism v7 software and Tukey’s multiple comparisons test.
